# Measuring Clinical Efficacy Through the Lens of Audit Data in Different Adult Eating Disorder Treatment Programmes

**DOI:** 10.3389/fpsyt.2020.599945

**Published:** 2020-12-01

**Authors:** Zhuo Li, Yasemin Dandil, Cindy Toloza, Anna Carr, Oyenike Oyeleye, Emma Kinnaird, Kate Tchanturia

**Affiliations:** ^1^Department of Psychological Medicine, King's College London, Institute of Psychology, Psychiatry and Neuroscience, London, United Kingdom; ^2^National Eating Disorders Service, South London and Maudsley NHS Foundation Trust, London, United Kingdom; ^3^Department of Psychology, Illia State University, Tbilisi, Georgia

**Keywords:** anorexia nervosa, inpatient, day treatment, efficacy, clinical significance, autism, comorbidity

## Abstract

**Background:** Audit data is important in creating a clear picture of clinical reality in clinical services, and evaluating treatment outcomes. This paper explored the data from an audit of a large national eating disorder (ED) service and evaluated the outcome of inpatient and day treatment programmes for patients with anorexia nervosa (AN) with and without autistic traits.

**Methods:** Four hundred and seventy-six patients receiving treatment for AN at inpatient (IP), day-care (DC) and step-up (SU) programmes were assessed at admission and at discharge on the following measures: autistic traits, body-mass-index (BMI), ED symptoms, depression and anxiety symptoms, work and social functioning, and motivation for change. Outcomes were analyzed first at a within-group level based on change in mean scores and then at an individual level based on the clinical significance of improvement in eating disorder symptoms. Outcomes were compared between patients with high autistic traits (HAT) and low autistic traits (LAT) in each programme.

**Results:** The findings suggest that 45.5% of DC and 35.1% of IP patients showed clinically significant changes in ED symptoms following treatment. Co-occurring high autistic traits positively predicted improvement in ED symptoms in IP setting, but was a negative predictor in DC. In IP, more HAT inpatients no longer met the BMI cut-off for AN compared to LAT peers. In terms of general psychopathology, patients with AN and HAT exhibited more severe depression symptoms, anxiety symptoms and social functioning impairment than their LAT peers, and these symptoms stayed clinically severe after treatment.

**Conclusions:** Patients with AN and hight autistic traits are more likely than their peers with low autistic traits to show weight restoration and improvement in ED systems after inpatient treatment. This reverses in DC, with high autistic trait patients less likely to improve after treatment compared to low autistic trait patients. Our results suggest that inpatient treatment with individualized and structured routine care may be an effective model of treatment for patients with AN and high autistic traits.

## Background

Anorexia nervosa (AN) is a severe eating disorder (ED) with a substantial death rate (1) that commonly develops around the ages of 16–17 (2). Practice guidelines in the United Kingdom (3), the United States of America (4) and across Europe (5) recommend inpatient treatment (IP) as the preferred treatment for moderately to severely ill patients with AN. However, with the accompanying financial costs and higher relapse rates (2, 6) of IP treatment, more recent studies have proposed that AN treatments may also be effective in other settings such as outpatient or day patient treatment. A multicentre randomized controlled trial (RCT) in 2014 (7) demonstrated the efficacy of outpatient treatment of AN to facilitate weight restoration and to reduce general and ED psychopathology. Similarly, another RCT on day patient treatment has shown that alongside effective weight restoration and maintenance, day treatment has an added advantage of being more cost-efficient, enabling patients to maintain their own social networks and transfer the skills they learn to day-to-day life more easily (8).

Subsequently, there has been a growing debate on whether day patient treatment can be superior to standardized IP treatment in providing more individualized and cost-effective treatment of AN (9). In this study, we present naturalistic clinical audit results and analyses of predictors of clinical significance from three treatment programmes in a specialist eating disorder service: inpatient (IP), step-up (SU) and day-care (DC). Each treatment programme incorporates an integrated and multidisciplinary approach to treatment, with each part of the service representing a different setting and intensity of intervention.

Although gathering practice-based evidence is equally important as evidence-based research in facilitating practice (10), the extent to which research evidence is valued among clinicians is variable. This is partly due to the perceived differences between clinical trial samples and the diversity of patients in clinical settings (11). To address the paucity of literature that evaluates ED treatment outcomes in naturalistic clinical settings, we used data from the clinical audit in an effort to reflect clinical reality.

Recent findings have also highlighted the over-representation of autism spectrum condition (ASC) in AN (12). Patients with comorbid ASC and AN tend to have longer durations of inpatient stay and poorer clinical outcomes upon discharge (13, 14), which highlights the need for tailored treatment interventions. Indeed, interviews with ASC patients with AN indicate that standard ED treatments do not meet the unique needs experienced by this population (15). Building on this growing research evidence, we further explored the influence of the comorbidity on treatment outcomes, given that patients in all three treatment groups were screened for autistic traits on admission to the service.

The aims of the current study are to add to the body of research by:

1. Reporting the treatment outcomes and predictors of clinically significant improvement in ED symptoms in inpatient and day treatment groups using a large audit data set from a national clinical ED service.
2. Exploring the prevalence of autistic traits in patients with AN and their influence on clinical outcomes.

## Methods

### Participants

**Figure 1:**
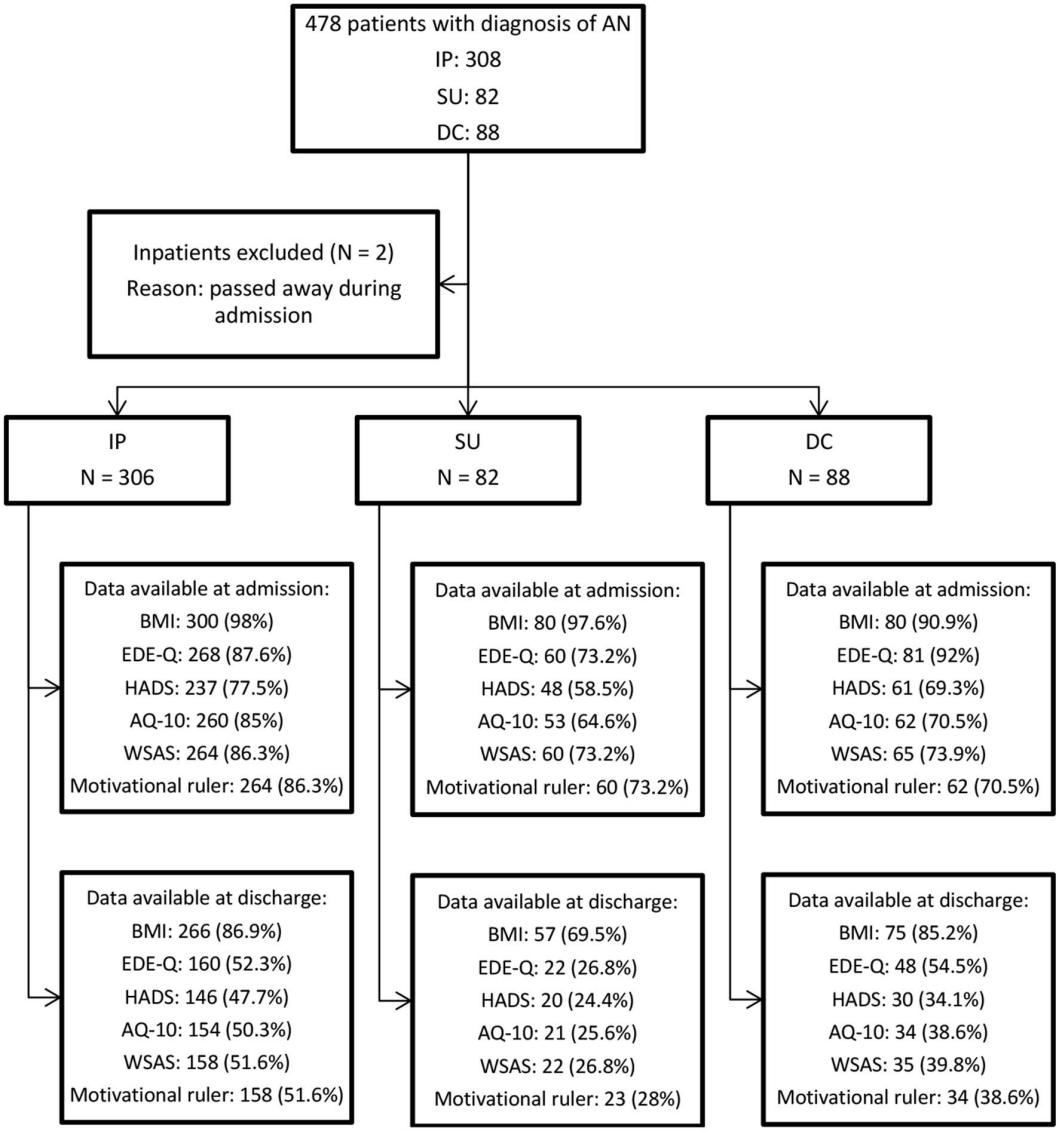
Sampling process flowchart. Data available at admission and discharge are reported in *N* of available response (response rate %).

We included a consecutive sample of patients who were admitted to the IP, SU and DC treatment groups at the National Eating Disorder Service between January 2012 and May 2020. Study permission was obtained from the Clinical Governance Committee Research and Development Office in 2004 and is ongoing. Patients completed a questionnaire pack which included clinical measures at the point of admission and discharge. Questionnaire response rates (Figure 1) were higher at admission than discharge due to some participants not returning questionnaires, refusal to complete certain parts of clinical measures, premature discharge or self-discharge from service. To reflect actual clinical practice, all patients with valid demographic data and complete or partly complete clinical measures were included in the study, with no replacement of missing values.

All included patients had a diagnosis of “AN restrictive type,” “AN binge-purge type” or “atypical AN” according to the 5th edition of the Diagnostic and Statistical Manual of Mental Disorders (DSM-5) (16). Due to small sample sizes, patients with other diagnoses were excluded from the analysis. During the study period, 308 IP, 82 SU and 88 DC patients completed the questionnaires upon admission. Two inpatients passed away during admission and were excluded from the analysis. Consequently, the final sample consisted of 476 patients with an AN diagnosis (Figure 1). Patients' sociodemographic and clinical characteristics are reported in Table 1.

**Table 1:** Sociodemographic and clinical characteristics at baseline.

	**IP (*N* = 306)**	**SU (*N* = 82)**	**DC (*N* = 88)**
**GENDER**			
Female	304 (99.3%)	78 (95.1%)	84 (95.5%)
Male	2 (0.7%)	4 (4.9%)	4 (4.5%)
**AGE**			
18–24 years	149 (48.7%)	32 (39%)	51 (58%)
25–29 years	63 (20.6%)	20 (24.4%)	20 (22.7%)
30–39 years	54 (17.6)	19 (23.2%)	11 (12.5%)
40 or above	40 (13%)	11 (13.4%)	6 (6.8%)
**ETHNICITY**			
White	271 (88.6%)	66 (80.4%)	80 (91%)
Black	2 (0.7%)	3 (3.7%)	–
Mixed	5 (1.6%)	4 (4.9%)	4 (4.5%)
Asian	14 (4.6%)	3 (3.7%)	3 (3.4%)
Other ethnic backgrounds	14 (4.6%)	6 (7.3%)	1 (1.1%)
Mean Age	28.1 (10.9)	29.5 (10.4)	25.4 (7.4)
BMI (kg/m^2^)	13.9 (1.3)	16.8 (2.1)	17.2 (1.7)
Duration of illness (years)	10.7 (9.4)	11 (9.3)	7.8 (7.8)
Duration of treatment (weeks)	15.9 (13.2)	20.4 (15)	27.4 (15.5)
**AN SUBTYPE**			
Restrictive	213 (69.6%)	59 (72%)	58 (65.9%)
Binge-Purge	80 (26.2%)	23 (28%)	30 (34.1%)
Atypical	13 (4.2%)	–	–
Living alone	52 (17%)	11 (13.4%)	7 (8%)
Living with parent/s	161 (52.7%)	29 (35.3%)	48 (54.5%)

*Data are mean (SD) or n (%)*.

### Treatment Programme Description

The primary focus of the IP included weight restoration, physical stabilization, psychological and occupational assessments and treatment based on formulation. To be eligible for the IP, patients have to be adults (>18 years) with BMI <15 and require refeeding. A multidisciplinary team of psychiatrists, psychologists, family therapists, nurses, medics, dieticians, occupational therapists, support workers and therapy enablers meet weekly to consult on cases and support each patient through their recovery journey. Individual therapy interventions include case formulation based on cognitive behavioral therapy [CBT; (17)], cognitive remediation therapy (CRT) adapted for AN (18), Cognitive Remediation and Emotion Skills Training (19), collaborative family work as well as carer support [for a detailed description of the inpatient group programme, see 2015 protocols by Tchanturia (20)]. All inpatients are also supported by highly specialized nurses, occupational therapist and dieticians with the re-feeding programme and individualized meal support.

The SU programme was introduced in April 2011 for patients who required support through the transition from IP and outpatient to the community. The SU programme operates 5 days a week, providing meal support and therapeutic group activities. Patients with BMI close to 15 and are medically stable to travel are eligible to attend. The multidisciplinary team at SU facilitates interventions that are mainly in groups where patients can interact with others, which assists with social skills training and motivation enhancement. Treatment mainly incorporates behavioral change strategies including positive thinking, outcome expectations, self-efficacy and concrete plans around occupation and living independently outside of the hospital.

The multidisciplinary team at DC helps patients move toward full recovery from their eating disorder, either as a step-down from intensive care or when outpatient treatment is not sufficient. The aim is for patients to maintain consistent weight restoration to a healthy BMI, in order to be able to engage in the group and individual psychological aspect of the programme. The DC programme operates 5 days a week, and only patients who are medically stable with BMI above 15 are eligible to attend. Interventions include CBT (17), cognitive analytic therapy [CAT; (21)], motivational enhancement therapy [MET; (22)], Maudsley model of anorexia nervosa treatment [MANTRA; (23)] and mentalisation-based therapy as well as occupational therapy groups such as well-being groups.

The day patient programmes DC and SU differ in terms of treatment scope and intensity. DC has a focus on full recovery and includes more psychological therapy sessions than SU, whereas SU is focused on group therapies and occupational therapy to help patients manage functioning outside of the hospital.

### Measures

The audit data consists of the following clinical measures:

*Eating Disorder Examination-Questionnaire (EDE-Q)* (24)

The EDE-Q is a widely used questionnaire that provides a comprehensive psychopathology assessment of eating-disordered behavior in a 36-item self-report format. The questionnaire is scored on four subscales: Restraint, Eating Concern, Shape Concern, and Weight Concern. A global EDE-Q score is calculated as the mean of the four subscales. Test–retest reliability ranges from 0.81 to 0.94 for the subscales (25), and 0.92 for the global score (26). In this study, Cronbach's alpha was 0.95 for IP and 0.96 for SU. For the DC sample audit, only global and subscale totals were reported whereas reply to each questionnaire item was not recorded, therefore we were not able to calculate Cronbach's alpha.

*Hospital Anxiety and Depression Scale (HADS)* (27)

The HADS is a 14-item self-rating instrument to assess the severity of anxiety and depression symptoms in a hospital setting, with the clinical cut-off at ≥10 for both depression and anxiety subscales. In this study, Cronbach's alpha was 0.89 for IP, 0.87 for DC, and 0.89 for SU.

*Work and Social Adjustment Scale (WSAS)* (28)

The WSAS is a five-item self-report measure of impaired work and social functioning which possibly affects areas of home management, ability to work, ability to pursue leisure activities (socially or privately) and ability to form and maintain close relationships. A higher score implies worse functioning: a WSAS score above 20 implies moderately severe or worse psychopathology. Scores between 10 and 20 are associated with less severe clinical symptoms. Scores below 10 are interpreted as subclinical. In this study, Cronbach's alpha was 0.83 for IP, 0.80 for DC and 0.92 for SU.

*Autism Spectrum Quotient, short version (AQ-10)* (29)

The AQ-10 is a 10-item, short version self-report questionnaire designed to screen for autistic traits in adults with average intelligence, with equivalent validity to the longer version of the AQ (29). Patients who score above the cut-off ≥6 are interpreted as having high levels of autistic traits, indicating a further ASC diagnostic assessment may be beneficial.

*Motivational ruler (MR)* (30)

The motivational ruler explores beliefs about the *importance* to change and the perceived *ability* to change. The ruler consists of two Likert scales ranging from 0 (not at all) to 10 (very much). Scores on both scales are combined into a “readiness to change score,” which is the mean score of importance and confidence to change.

### Statistical Analysis

The outcome measures were obtained shortly after admission in the treatment programme and close to discharge, including BMI, EDE-Q, HADS, AQ-10, WSAS and motivational ruler. Results were analyzed at both group level and individual level. First, due to heterogeneity in symptom severity among different treatment cohorts, within-group analysis was conducted for each treatment setting. All outcome data was tested with the Shapiro-Wilk test of normality and repeated measures *t*-tests or Wilcoxon Signed-rank tests were performed where applicable.

Second, EDE-Q outcomes were evaluated on an individual level for all patients using the criteria of clinical significance (31), which allocates each patient into one of four groups based on whether there was a “significant and reliable” decrease in EDE-Q global scores (measured by Jacobson and Truax's Reliable Change Index (RCI) (31)) and whether the post-treatment EDE-Q is returned to the normative range of functioning observed in the U.K. population (32): (1) “Recovered”: Individuals who showed significant pre-post change with post-treatment scores in the functional range; (2) “Improved but not recovered”: Individuals who showed statistically significant change after treatment, yet post-treatment EDE-Q scores still indicated high levels ED symptomatology (3) “Unchanged/Undetermined”: Patients who fell within the functional EDE-Q range of the population at the end of treatment, but their change was not statistically reliable; and (4) “Reliably deteriorated”: These patient's post-treatment EDE-Q scores indicate high levels of ED symptomatology and have significantly deteriorated compared to pre-treatment scores. Clinical significance for individual patients was calculated based on the established EDE-Q norms among adults in the U.K. (32) and the reported test-retest reliability of the EDE-Q scale (11).

Furthermore, binary logistic regression analysis was performed to identify predictors of a clinically significant improvement in EDE-Q Global score for IP and DC. The dependant variable was set dichotomously between “Recovered/Improved” vs. “Unchanged/Deteriorated.” Potential predictor variables that have been ascertained by previous studies and that were available at admission in our study were added to the regression analysis, including age at admission, clinical characteristics (purging subtype, duration of illness, high autistic traits reflected by an AQ-10 score above 6, BMI at admission, length of admission) and baseline scores (HADS anxiety subscale, HADS depression subscale, WSAS total, readiness to change, EDE-Q global). EDE-Q subscale scores showed significant multicollinearity (VIF > 5) therefore EDE-Q global was included instead in the regression analysis.

To further investigate the prevalence and influences on clinical outcomes of high autistic traits in AN patients, we separated patients in each treatment group further into a high autistic traits (HAT) and low autistic traits (LAT) groups based on their baseline score on the AQ-10 (HAT group scores ≥6). We first screened the data for normality before testing the difference in clinical outcomes between HAT and LAT patients in each treatment programme. Independent samples *t*-tests and Mann-Whitney *U* tests were performed for all clinical measures depending on the normality of the data.

The Bonferroni correction was used for multiple-comparison correction. Effect sizes were reported in Cohen's *d* for *t*-tests with two groups of the same size or Hedge's *g* to control for sample size, matched pairs rank biserial correlation for Wilcoxon signed-rank tests (33), and point biserial correlation *r* for Mann-Whitney U tests (34). For the Cohen's *d* and Hedge's *g* effect sizes calculated, values around 0.2 were considered as small, 0.5 as medium, and 0.8 as large (35). For biserial correlations, values of 0.37, 0.24, and 0.10 represented large, medium and small as suggested by McGrath and Meyer (34). G^*^Power 3.1 was used to perform power analysis and calculate the minimal detectable effect (36).

## Results

The final sample consisted of 476 patients aged between 18 and 69 years (Figure 1; Table 1). Mean duration of treatment was 15.9 weeks for inpatients, 20.4 weeks for SU and 27.4 weeks for DC patients. Clinical outcomes of included patients are presented in Tables 2A,B. For each clinical measure, only participants who had completed the corresponding questionnaire at both admission and discharge are included in the analysis. In both IP and SU, there were no significant differences between those for whom all data were available and those for whom data were partly unavailable in terms of age, BMI at admission, length of admission, duration of illness, and BMI change during treatment (Supplementary Table 1). In DC, length of admission was significantly longer for patients with all data available (mean = 36.8 weeks, SD = 14.5 weeks) compared to those with incomplete data (mean = 23.6 weeks, SD = 14.3 weeks, *t*(81) = 3.79, *p* < 0.001); there were no differences in other demographic characteristics between these two groups.

**Table 2A:** Admission and discharge outcome comparison for IP, SU, and DC.

		**Inpatient *N* = 306**	**Step-up *N* = 82**	**Day-Care *N* = 88**
BMI	Admission^a^	13.90 (1.34)	16.76 (2.10)	17.10 (1.65)
	Discharge^a^	16.08 (1.88)	16.96 (2.28)	18.59 (2.17)
	*t* (df)	23.31 (265)	1.38 (56)	7.3 (73)
	*p*-value	<0.001	0.174	<0.001
	ES^b^	1.34	0.09	0.77
	Power	1.00	0.09	1.00
EDE-Q global	Admission	4.13 (1.58)	3.57 (1.71)	4.08 (1.24)
	Discharge	3.02 (1.58)	3.34 (1.72)	3.09 (1.55)
	*t* (df)	10.45 (150)	1.13 (17)	5.53 (43)
	*p*-value	<0.001	0.27	<0.001
	ES	0.70	0.13	0.71
	Power	1.00	0.05	0.99
HADS-Anxiety	Admission	15.06 (4.31)	13.27 (4.54)	14.06 (3.54)
	Discharge	12.54 (4.63)	12.73 (4.13)	12.32 (4.41)
	*t* (df)	7.29 (127)	0.51 (14)	2.64 (24)
	*p*-value	<0.001	0.62	0.014
	ES	0.56	0.12	0.44
	Power	1.00	0.01	0.40
HADS-Depression	Admission	12.05 (4.91)	9.14 (4.80)	10.44 (4.19)
	Discharge	8.02 (4.80)	7.64 (4.70)	7.84 (4.73)
	*t* (df)	9.71 (127)	1.67 (13)	3.42 (24)
	*p*-value	<0.001	0.12	0.002
	ES	0.83	0.32	0.58
	Power	1.00	0.11	0.68
WSAS	Admission	26.82 (8.60)	26.50 (11.15)	23.69 (9.15)
	Discharge	21.58 (10.03)	20.55 (11.05)	18.19 (8.07)
	*t* (df)	6.84 (147)	–^*^	4.29 (26)
	*p*-value	<0.001	–^*^	<0.001
	ES	0.56	0.63^**^	0.64
	Power	1.00	0.16	0.90
AQ-10	Admission	4.24 (2.46)	4.91 (1.77)	4.67 (2.13)
	Discharge	3.70 (2.32)	4.38 (1.69)	3.56 (2.04)
	*t* (df)	3.47 (142)	–^*^	2.14 (26)
	*p*-value	0.001	–^*^	0.042
	ES	0.23	0.67^**^	0.53
	Power	0.77	0.14	0.24
Motivational Ruler (“Readiness to change”)	Admission	6.37 (2.24)	6.03 (2.66)	7.36 (1.52)
	Discharge	7.14 (2.19)	6.68 (2.51)	7.50 (1.39)
	*t* (df)	4.43 (148)	1.79 (18)	0.47 (24)
	*p*-value	<0.001	0.09	0.642
	ES	0.35	0.25	0.10
	Power	0.95	0.14	0.01

*EDE-Q global, Eating Disorder Examination Questionnaire global subscale; HADS, Hospital Anxiety and Depression Scale; WSAS, Work and Social Adjustment Scale; AQ-10, Autism Spectrum Quotient-−10 items*.

a *Pre- and post-assessment data are reported in mean (M) and standard deviation (SD)*.

b *ES: Effect size (Cohen's d unless otherwise specified)*.

* *Wilcoxon signed-rank test results reported in Table 2B*.

** *Matched pairs rank biserial reported as effect size because Wilcoxon signed-rank test was used*.

**Table 2B:** Changes in WSAS and AQ-10 pre-post measures for SU based on Wilcoxon signed-rank test.

	**Admission**	**Discharge**								
	**Mean**	**25th pctl**.	**50th pctl**.	**75th pctl**.	**Mean**	**25th pctl**.	**50th pctl**.	**75th pctl**.	***Z***	***p***
WSAS	26.50 (11.15)	17.0	28.5	37.0	20.55 (11.05)	9.8	23.5	28.5	−2.106^a^	0.035
AQ	4.91 (1.77)	4.0	5.0	6.0	4.38 (1.69)	3.0	4.0	6.0	−1.732^a^	0.083

*WSAS, Work and Social Adjustment Scale; AQ, Autism Spectrum Quotient*.

a *Based on positive ranks*.

### Clinical Outcomes

BMI

BMI significantly increased for IP and DC patients (Table 2A). Mean BMI for IP patients increased from 13.90 (“severe AN” according to the DSM-IV BMI cut-off) to 16.08 (“AN”) upon discharge, with an effect size larger than one standard deviation (Cohen's *d* = 1.34). For SU, the change in BMI was not statistically significant. Mean BMI for DC patients increased from 17.10 (“AN” on the Maudsley BMI table) to 18.59 (“underweight”), with a medium to large effect size of 0.77.

*EDE-Q global score* (24)

Bonferroni corrected tests revealed that both IP and DC patients showed statistically significant improvement in EDE-Q global score upon discharge (Table 2A). The effect size was medium to large for both groups (0.70 for IP and 0.71 for DC). There was no significant change in the mean EDE-Q global score in SU patients.

Regarding outcomes of clinical significance for improvement in the EDE-Q global score (Table 3), 45.5% of DC patients and 35.1% of IP patients were in the “Recovered” or “Improved” categories, whereas 52.3% of DC, 62.9% of IP, and 83.3% of SU patients remained unchanged in eating disorder psychopathology after treatment.

**Table 3:** Clinical significance outcomes for the EDE-Q global score.

	**Inpatient**	**Step-up**	**Day-Care**
Recovered/Improved	53 (35.1%)	2 (11.1%)	20 (45.5%)
Unchanged	95 (62.9%)	15 (83.3%)	23 (52.3%)
Deteriorated	3 (2.0%)	1 (5.6%)	1 (2.3%)

*EDE-Q global, Eating Disorder Examination Questionnaire global subscale*.

Results of binary logistic regression analysis (Table 4) showed that for IP, predictors of a clinically significant improvement in ED symptoms include a higher self-reported EDE-Q global score at admission and high autistic traits (Nagelkerke's *R*^2^ = 19.6%). For DC, the predictors include a higher “readiness to change” reflected by the motivational ruler and lower autistic traits (AQ-10 < 6) at baseline (Nagelkerke's *R*^2^ = 44.3%). For SU, no regression model was generated due to the low number of observations.

**Table 4:** Significant predictors of a clinically significant change in eating disorder symptoms (EDE-Q Global score) in IP and DC.

	**B**	**SE**	**Wald**	***P***	**EXP (B)**	**95% confidence interval for EXP(B)**	
						**Lower bound**	**Upper bound**
**Inpatient**							
EDE-Q global	0.59	0.22	7.11	0.008	1.81	1.17	2.80
Autistic traits (AQ-10 ≥ 6)	0.97	0.49	3.90	0.048	2.64	1.01	6.90
**Day-Care**							
Readiness to change (MR)	1.22	0.53	5.33	0.021	3.40	1.20	9.60
Autistic traits (AQ-10 ≥ 6)	−3.06	1.54	3.95	0.047	0.047	0.00	0.96

*EDE-Q global, Eating Disorder Examination Questionnaire global subscale; AQ-10, Autism Spectrum Quotient-−10 items; MR, Motivational Ruler*.

*HADS* (27)

Bonferroni corrected tests revealed that the IP patients showed statistically significant improvement (Table 2A) in both anxiety symptoms (*t*(127) = 7.29, *p* < 0.001, Cohen's *d* = 0.56) and depression symptoms (*t*(127) = 9.71, *p* < 0.001, Cohen's *d* = 0.83). SU patients did not show statistically significant improvement on depression (*p* = 0.12, Cohen's *d* = 0.32) or anxiety subscale (*p* = 0.62, Cohen's *d* = 0.12). DC patients showed significant improvement in depression but not in anxiety symptoms (Depression: *t*(24) = 3.42, *p* = 0.002, Cohen's *d* = 0.58; Anxiety: *t*(24) = 2.64, *p* = 0.014, Cohen's *d* = 0.44).

*WSAS* (28)

All three treatment groups showed statistically significant improvements on the work and social functioning scale (Tables 2A,B). Mean WSAS total score decreased for IP from 26.82 (SD = 8.6) to 21.58 (SD = 10.03) on discharge, from 23.69 (SD = 9.15) to 18.19 (SD = 8.07) for DC, and from 26.50 (SD = 11.15) to 20.55 (SD = 11.05) for SU. The effect size of change in WSAS outcome for IP, DC and SU was 0.56, 0.64, and 0.63 respectively.

*AQ-10* (29)

IP patients showed a statistically significant decrease in AQ-10 score from 4.24 (SD = 2.46) to 3.70 (SD = 2.32) upon discharge (Cohen's *d* = 0.23) (Table 2A). Although there was no statistically significant change in AQ-10 for SU patients, the effect size of the pre-post difference was medium to large (*Z* = −1.732, *p* = 0.083, ES = 0.67) (Table 2B). No significant decrease in AQ-10 was observed in DC patients although the effect size was medium (Cohen's *d* = 0.53).

*Motivational Ruler* (30)

IP patients showed statistically significant improvement in “readiness to change” score, from 6.37 (SD = 2.24) at admission to 7.14 (SD = 2.19) at discharge, and the effect size was small to large (Cohen's *d* = 0.35). DC patients' score on the motivational ruler remained high from 7.36 (SD = 1.52) at admission to 7.50 (SD = 1.39) discharge (Cohen's *d* = 0.10) and with no significant change. SU patients did not show statistically significant improvement and the effect size of the pre-post difference was small (Cohen's *d* = 0.25).

### High Autistic Traits and AN Comorbidity

#### Prevalence and Baseline Characteristics

Autistic traits were assessed on admission using the Autism Quotient short screening tool (AQ-10). As a result, 28.1% of IP patients (*N* = 86, 85 female, one male), 24.4% of SU patients (*N* = 20, one male, 19 female) and 22.7% of DC patients (*N* = 20, all female) scored above the clinical cut-off of six, forming the HAT group.

Baseline characteristics of patients with and without autistic traits are reported in Supplementary Table 2. In IP, HAT and LAT patients did not differ in demographics such as age, age of onset, duration of illness and living arrangements. HAT inpatients had a significantly higher BMI on admission (mean = 14.22, SD = 1.44) than LAT inpatients (mean = 13.74, SD = 1.31, *t*(252) = −2.6, *p* = 0.009). In terms of clinical characteristics, HAT inpatients scored significantly higher on all clinical variables and lower on readiness to change.

In SU, no significant differences in demographics were found between HAT and LAT patients. HAT patients had more severe anxiety symptoms at baseline (*p* = 0.009) compared to the LAT group.

In DC, HAT patients were significantly younger (*p* = 0.014) with shorter duration of illness than LAT patients (*p* = 0.014). HAT patients were also more likely to live with family (*p* = 0.013) and showed more severe social functioning problems (*p* = 0.02) at baseline.

#### Influence on Clinical Outcomes

As presented in Table 5, discharge BMI of HAT inpatients was higher than LAT inpatients but this difference was not significant after Bonferroni correction (*p* = 0.013, Hedge's *g* = 0.35; *post-hoc* power calculations revealed that the IP sample size was adequate to detect medium to large effects (*f* = 0.39, *p* < 0.05, power = 0.80)). About a third (30.3%) of HAT inpatients no longer met the DSM-IV BMI cut-off for AN (BMI < 17.5) at the end of the treatment, whereas this was only achieved by 19.5% of LAT inpatients (Fisher's exact *p* = 0.05). Meanwhile, HAT inpatients also scored higher with moderate effect size on the anxiety subscale (*p* = 0.013, Hedge's *g* = 0.46), depression subscale (*p* = 0.009, biserial correlation *r* = 0.22), and on work and social functioning (*p* = 0.008, biserial correlation *r* = 0.22) after treatment. In particular, HAT inpatients' scoring on anxiety and work and social functioning impairment on discharge were well above the threshold for clinically severe symptoms after inpatient treatment.

**Table 5:** Clinical outcome data on discharge of patients with high (HAT) and low (LAT) autistic traits.

	**IP**				**SU**				**DC**			
	**HAT**	**LAT**	***P***	**ES (power)**	**HAT**	**LAT**	***p***	**ES (power)**	**HAT**	**LAT**	***p***	**ES (power)**
BMI	16.60 (2.00)	15.94 (1.84)	0.013	0.35 (0.41)	16.73 (2.27)	16.89 (2.69)	0.85	0.06 (0.01)	19.04 (2.37)	18.66 (1.62)	0.49	0.20 (0.02)
EDE-Q global	3.34 (1.53)	2.87 (1.61)	0.094^*^	0.14^**^ (0.17)	3.82 (1.91)	3.01 (1.70)	0.38	0.46 (0.02)	2.96 (1.58)	3.43 (1.43)	0.39	0.32 (0.03)
HADS-Anxiety	14.02 (4.27)	11.87 (4.87)	0.013	0.46 (0.50)	15.67 (3.78)	11.90 (3.60)	0.07	1.03 (0.12)	12.75 (5.01)	12.19 (4.39)	0.78	0.12 (0.01)
HADS-Depression	9.56 (4.22)	7.40 (4.89)	0.009^*^	0.22^**^ (0.48)	9.67 (5.68)	8.10 (4.53)	0.55	0.32 (0.02)	8.44 (3.81)	7.60 (5.44)	0.69	0.17 (0.01)
WSAS	24.55 (8.84)	19.70 (10.42)	0.008^*^	0.22^**^ (0.59)	24.83 (12.32)	17.73 (9.86)	0.21	0.66 (0.05)	19.56 (6.25)	17.95 (8.90)	0.63	0.20 (0.02)
Readiness to change	6.61 (2.59)	7.34 (1.96)	0.197^*^	0.11^**^ (0.17)	5.33 (3.49)	7.32 (1.91)	0.15	0.78 (0.05)	7.61 (1.11)	7.42 (1.49)	0.73	0.14 (0.01)

*EDE-Q global, Eating Disorder Examination Questionnaire global subscale; HADS, Hospital Anxiety and Depression Scale; WSAS, Work and Social Adjustment Scale. All baseline data reported in mean (SD); ES = Effect size. Unless otherwise specified, p-values for independent samples tests and Hedge's g effect sizes are reported*.

Exceptions:

* *Mann-Whitney U test p-value reported*.

** *Effect size is reported in point biserial correlation r*.

In SU, 25% of HAT patients and 29.1% of LAT patients no longer met the BMI cut-off for AN and the between-group difference was not significant (Fisher's exact *p* = 0.53). SU HAT patients showed statistically insignificant but substantially more severe anxiety symptoms (*p* = 0.07, Hedge's *g* = 1.03), work and social functioning impairment (*p* = 0.21, Hedge's *g* = 0.66) and lower readiness to change (*p* = 0.15, Hedge's *g* = 0.78) upon discharge. *Post-hoc* power analysis revealed that the SU sample size was only adequate to detect large effects (*f* = 0.98, *p* < 0.05, power = 0.80).

In DC, no significant difference was found between HAT and LAT patients on all clinical measures, and 77.8% of HAT and 77.8% of LAT patients no longer met the BMI cut-off for AN.

## Discussion

Our results indicated that IP, DC, and SU all achieved significant clinical outcomes that were in line with each group's focus of treatment. Although we are not able to statistically compare the scale of increase in BMI between IP and DC due to the differences in clinical characteristics between the groups, patients in both groups showed substantial and significant weight restoration after treatment. The effect size of ES = 0.77 for the increase in BMI in DC patients is similar to the result of recent outpatient treatment RCT (7) which reported ES = 0.62 to 1. The high effect size of ES = 1.34 for BMI increase in IP is comparable with meta-analytically calculated effect size of inpatient AN treatment with ES = 1.19 (CI: 1.07–1.30) (37). Patients in both DC and IP also improved significantly in anxiety, depression symptoms as well as work and social functioning. Currently, conclusive literature on the clinical efficiency of day treatment or partial hospitalization model is still limited. The strong outcomes of DC treatment we present can pave way for future controlled study to investigate day treatment as an efficient and cost-effective substitute for inpatient treatment.

Because the focus of SU treatment was relapse prevention instead of psychopathological treatment, SU patients' BMI and psychopathology outcomes were not significantly improved. Instead, the SU group experienced a substantial and significant improvement in work and social functioning. This may reflect the fact that the SU treatment was much more group-centrered than the other two types of treatment and the occupational therapy led programme supported patients to function outside of the hospital, focusing more on social skills training and motivation enhancement. Preliminary findings using Work and Social Adjustment Scale (WSAS) in patients with AN have found that scores were unrelated to BMI but significantly correlated with the severity of ED symptoms (38). Improvement in WSAS, therefore, may play a vital role in determining the patient's stage of recovery.

We found that both IP and SU patients experienced a reduction in AQ-10 scores following treatment. This decrease could suggest that these scores reflect features associated with AN, and so improve with treatment, rather than underlying ASC. Studies have highlighted problems of socio-cognitive disturbance in patients with AN that are similar to autistic traits (39, 40), and it is worth noting that some of the traits measured by the AQ-10 are also observed in AN patients [i.e., cognitive inflexibility (41), social difficulties (42)]. Therefore, the decrease in AQ-10 scores could be a result of patients' improvement in social and cognitive functioning. We also assessed the influence of autistic traits on the clinical significance of treatment outcomes. In the regression analysis of factors predicting clinically significant improvement in ED symptoms, high autistic traits appeared to be a positive predictor in IP setting but a negative predictor in DC setting, suggesting that patients with high traits are more likely to significantly improve in ED symptoms than their peers with low traits when treated in IP, but the situation reverses in DC. There are two possible explanations: first, IP treatment was more individualized, and each patient was supported daily by highly specialized nurses, creating an environment that is potentially safer for patients with the comorbidity. DC treatment on the other hand required greater amounts of group participation, which means that individuals with high autistic traits who find social interaction and social-emotional understanding difficult (43, 44) are more likely to face additional challenges. As a result, this may have contributed to the lack of improvement in ED symptoms for patients with high traits. Secondly, a fundamental part of DC treatment includes holding boundaries (i.e., weight, attendance and group contribution) that patients need to adhere to otherwise they will be discharged. As a result of such strict boundaries in order to facilitate recovery, individuals with high autistic traits may have found this difficult due to their heightened levels of cognitive rigidity and strong preference for their own sets of routines (45). This finding highlights the need to consider the presence of autistic traits when formulating models of treatment and the importance of individualized intensive care when treating these patients, particularly in day treatment settings.

Among other predictors, DC patients with higher motivation were more likely to achieve clinically significant improvement in eating disorder symptoms, while a higher baseline EDE-Q global score was a positive predictor for IP. The latter was a counterintuitive finding, given that better treatment outcome tends to be associated with less severe baseline ED symptoms (46, 47). It is possible that patients who were more transparent when completing the self-reported questionnaire were more prepared and willing to cooperate during treatment. Similarly, HAT patients with EDs (whose self-reported EDE-Q scores on admission were significantly higher than LAT patients) may have added to the predicting power of EDE-Q global score of clinical significance, since HAT patients were more likely to achieve clinically significant improvement in the IP setting.

Finally, it was found that social functioning of LAT patients was restored below clinical severity (WSAS threshold = 20) upon discharge. However, HAT patients showed difficulties in social functioning that were maintained above clinical severity even after treatment in both IP and SU, an outcome that is close to the level of functional difficulties observed in female patients with severe OCD (mean WSAS = 26.2, SD = 9.2) (48). In DC, baseline WSAS scores of HAT patients were just as severe as in the other two treatment groups but were restored to below 20 after treatment. This may be due to the higher BMI of DC patients, as previous research has found that lower BMI was a significant predictor of work and social adjustment problems (49). Furthermore, HAT patients with AN were also more likely to exhibit severe depression symptoms, anxiety symptoms and social functioning impairment on admission, and these symptoms tend to stay clinically severe after treatment. This is in line with previous research which found that autistic people with AN are at risk of poorer illness outcomes (13, 50), highlighting the need for adapted treatments for this population.

A limitation in this study is the lack of randomisation and an absence of a control group. The study was based on naturalistic observations, therefore it is impossible to state unequivocally that the observed outcomes were due to treatment. In an effort to compensate for this limitation, the most stringent criteria for evaluating clinical significance was used. Second, the evaluation of autistic traits in this study were based on the patient's score on the AQ-10, a measure of self-reported autistic traits instead of a formal diagnosis of autism. Significantly, the AQ-10 may lack validity in clinical samples with high levels of autistic traits and high levels of anxiety, as seen in AN populations (51–53). Future studies with participants that have received a formal diagnosis can help elucidate the relationship between autism, AN and treatment outcomes. Furthermore, patient's completion of the questionnaire pack was not compulsory, and our results may only represent those who were willing and able to fill in the questionnaires. This resulted in a high loss to follow-up and a lack of statistical power especially for SU. Meanwhile, our data is still useful in exploring clinical reality and outcomes of AN patients in a naturalistic clinical setting. Lastly, clinical outcome for all treatment groups would be better evaluated with follow up analysis investigating the likelihood of relapse, improvement in employment status and stabilization in BMI. Further follow up studies to assess clinical outcomes are needed.

## Conclusions

Our data suggests that although inpatient and day treatment groups all achieved significant outcomes that were in line with each group's treatment focus, for patients with AN and high autistic traits, inpatient treatment with individualized care may be a preferred model of treatment that leads to better outcomes in treating ED symptoms. Future studies on more effective treatment adaptations for patients with the comorbidity are of immense practical relevance.

## Data Availability Statement

The datasets generated for this article are not readily available because no such consent was obtained from the patients. Anonymised and analyzed data results are available from the corresponding author on reasonable request. Requests to access the datasets should be directed to Kate Tchanturia, kate.tchanturia@kcl.ac.uk; Zhuo Li, zhuo.li@kcl.ac.uk.

## Ethics Statement

The studies involving human participants were reviewed and approved by the Clinical Governance Committee Research and Development Office in South London and Maudsley NHH Trust in 2004. All procedures were according to the latest version of the Declaration of Helsinki. Written informed consent for participation was not required for this study in accordance with the national legislation and the institutional requirements.

## Author Contributions

ZL, KT, YD, CT, and AC: conceptualization. ZL: formal analysis and writing—original draft preparation. YD, ZL, AC, and CT: investigation and data curation. ZL, YD, OO, CT, AC, EK, and KT: writing—review and editing. KT: supervision and funding acquisition. All authors have read and agreed to the published version of the manuscript. All authors contributed to the article and approved the submitted version.

## Conflict of Interest

The authors declare that the research was conducted in the absence of any commercial or financial relationships that could be construed as a potential conflict of interest.
