# Supplementary material for: Measuring Clinical Efficacy Through the Lens of Audit Data in Different Adult Eating Disorder Treatment Programmes
Source: Front Psychiatry. 2020 Dec 1;11:599945. doi: 10.3389/fpsyt.2020.599945 (PMC7736031; doi:10.3389/fpsyt.2020.599945)
Supplement: Supplementary file 1 [file Data_Sheet_1.docx]

Supplementary Material

**Supplementary Table S1. Comparison of clinical characteristics between patients with complete and incomplete assessment data**

|  | **IP** |  |  | **SU** |  |  | **DC** |  |  |
| --- | --- | --- | --- | --- | --- | --- | --- | --- | --- |
|  | Patients with complete data | Patients with incomplete data | Test statistics | Patients with complete data | Patients with incomplete data | Test statistics | Patients with complete data | Patients with incomplete data | Test statistics |
| **Age, years** | 26.9 (9.5) | 28.9 (11.7) | *t*_304_ = 1.60  *P* = .11 | 26.1 (7.1) | 30.2 (10.9) | *t*_80_ = 1.32  *P* = .19 | 25.5 (6.8) | 25.3 (7.6) | *t*_86_ = .12  *P* = .91 |
| **BMI at admission** | 13.93 (1.34) | 13.89 (1.39) | *t*_296_ = .27  *P* = .79 | 16.29 (1.47) | 16.80 (2.16) | *t*_78_ = .83  *P* = .41 | 17.77 (1.90) | 16.98 (1.55) | *t*_78_ = 1.97  *P* = .053 |
| **Change in BMI** | 2.37 (1.24) | 2.03 (1.71) | *t*_264_ = 1.84  *P* = .07 | .52 (.86) | .11 (1.16) | *t*_55_ = 1.17  *P* = .25 | 1.81 (1.67) | 1.35 (1.80) | *t*_72_ = 1.05  *P* = .30 |
| **Length of admission (weeks)** | 15.4 (9.3) | 16.3 (15.3) | *t*_303_ = .58  *P* = .56 | 22.2 (16.0) | 17.2 (12.8) | *t*_58_ = 1.17  *P* = .25 | 36.8 (14.5) | 23.6 (14.3) | *t*_81_ = 3.79  *P* < .001 |
| **Duration of illness** | 9.5 (8.8) | 11.7 (9.8) | *t*_259_ = 1.91  *P* = .06 | 7.3 (5.4) | 12.1 (10.0) | *t*_58_ = 1.7  *P* = .09 | 7.4 (7.7) | 7.9 (7.8) | *t*_82_ = .29  *P* = .77 |

*data reported in (mean (SD)) unless otherwise specified.

**Supplementary Table S2.** Baseline sociodemographic and clinical characteristics of HAT and LAT patients

|  | **IP** |  |  | **SU** |  |  | | **DC** | |  | |  | |  |
| --- | --- | --- | --- | --- | --- | --- | --- | --- | --- | --- | --- | --- | --- | --- |
|  | HAT | LAT | Test statistics | HAT | LAT | Test statistics | | HAT | | LAT | | Test statistics | |  |
| **Age, years** | 27.08 (9.26) | 27.43 (9.7) | *t*_258_ = .28  *P* = .78 | 28.05 (8.91) | 28.27 (8.19) | *t*_51_ = .093  *P* = .93 | | 22.25 (3.52) | | 25.74 (7.37) | | *t*_60_ = 2.52  *P* = .014 | |  |
| **Age at onset** | 16.25 (4.34) | 16.97 (6.97) | *t*_235_ = .83  *P* = .41 | 17.16 (8.7) | 16.93 (5.17) | *t*_47_ = -.11  *P* = .91 | | 19.56 (2.28) | | 21.03 (5.81) | | *t*_51_ = 1.34  *P* = .19 | |  |
| **Duration of illness** | 10.74 (8.91) | 10.33 (8.75) | *t*_237_ = -.34  *P* = .73 | 11 (9.94) | 11.57 (8.38) | *t*_47_ = .21  *P* = .83 | | 5.1 (3.34) | | 8.84 (8.21) | | *t*_58_ = 2.52  *P* = .014 | |  |
| **BMI** | 14.22 (1.44) | 13.74 (1.31) | *t*_252_ = -2.6  *P* = .009 | 16.85 (1.93) | 16.65 (2.55) | *t*_51_ = -.3  *P* = .77 | | 17.68 (1.87) | | 17.25 (1.48) | | *t*_54_ = -.95  *P* = .35 | |  |
| **Living arrangements** (n (%)) | | |  |  |  | |  | |  | |  | |  | |
| Family | 53 (63.1%) | 99 (60.4%) | *χ*^2^ = .17  *P* = .68 | 10 (52.6%) | 15 (46.9%) | *χ*^2^ = .16  *P* = .69 | | 15 (83.3%) | | 20 (48.8%) | | *χ*^2^ = 6.19  *P* = .013 | |  |
| Partner/cohabiting | 12 (14.3%) | 38 (23.2%) | *χ*^2^ = 2.72  *P* = .10 | 5 (26.3%) | 15 (46.9%) | *χ*^2^ = 2.11  *P* = .15 | | 3 (16.7%) | | 17 (41.5%) | | *χ*^2^ = 3.43  *P* = .064 | |  |
| Alone | 19 (22.6%) | 27 (16.5%) | *χ*^2^ = .17  *P* = .68 | 4 (21.1%) | 2 (6.3%) | *χ*^2^ = 2.52  *P* = .11 | | 0 | | 4 (9.8%) | | *--* | |  |
| **EDE-Q global** | 4.59 (1.48) | 3.77 (1.7) | *t*_190_ = -3.98  *P* < .001 | 3.87 (2.06) | 3.53 (1.74) | *t*_51_ = -.64  *P* = .53 | | 4.15 (1.37) | | 4.14 (1.26) | | *t*_59_ = -.03  *P* = .98 | |  |
| **HADS-Anxiety** | 16.53 (3.91) | 13.42 (4.81) | *t*_176_ = -5.26  *P* < .001 | 16.47 (3.52) | 13.16 (4.23) | *t*_46_ = -2.74  *P* = .009 | | 15.1 (2.95) | | 13.96 (4.58) | | *t*_56_ = -1.01  *P* = .32 | |  |
| **HADS-Depression** | 13.58 (4.51) | 10.71 (5.13) | *t*_230_ = -4.15  *P* < .001 | 12.06 (4.9) | 10.07 (4.91) | *t*_46_ = -1.36  *P* = .18 | | 11.76 (4.58) | | 10.43 (4.37) | | *t*_55_ = -1.06  *P* = .29 | |  |
| **WSAS** | 29.68 (6.85) | 24.89 (10.04) | *t*_227_ = -4.45  *P* < .001 | 29.75 (10.66) | 23.7 (11.47) | *t*_51_ = -1.91  *P* = .062 | | 28.48 (6.56) | | 23.08 (9.35) | | *t*_58_ = -2.31  *P* = .02 | |  |
| **Readiness to change** | 5.62 (2.3) | 6.55 (2.33) | *t*_252_ = 2.99  *P* = .003 | 5.2 (2.48) | 6.46 (2.4) | *t*_51_ = 1.83  *P* = .07 | | 7.42 (1.26) | | 6.98 (1.6) | | *t*_55_ = -1.05  *P* = .3 | |  |

*Baseline data reported in (mean (SD)) unless otherwise specified.
